# Effectiveness of music-based interventions for cognitive rehabilitation in Parkinson’s disease: a systematic review of randomized controlled clinical trials

**DOI:** 10.1186/s41155-023-00259-x

**Published:** 2023-08-10

**Authors:** Leonardo Francisco Citon, Amer Cavalheiro Hamdan

**Affiliations:** 1 Graduate Program in Psychology, Federal University of Paraná, Curitiba, Brazil; 2 Department of Psychology, Federal University of Paraná, Curitiba, PR 80020-300 Brazil

**Keywords:** Parkinson’s disease, Music-based interventions, Music therapy, Cognitive rehabilitation, Cognition

## Abstract

**Background:**

Music-based interventions are promising for cognitive rehabilitation in Parkinson’s disease; however, systematic reviews covering the topic are scarce.

**Objective:**

To analyze the effectiveness of music-based interventions for cognitive rehabilitation in PD.

**Method:**

Systematic review study based on PRISMA criteria. The descriptors Parkinson’s disease, Parkinson’s disease, idiopathic Parkinson’s disease, music-based interventions, music therapy, music training, auditory stimulation, music, rhythm, rhythmic, cognition, and cognitive were used. Five databases were searched PubMed/MEDLINE, PsycInfo, Scopus, Web of Science, and Cochrane in May 2022. Only randomized controlled trials with no limit on publication date or language were included. Risk of bias was assessed following Cochrane Collaboration criteria for development of systematic intervention reviews.

**Results:**

Nine hundred nineteen articles were found by the descriptors; 266 were excluded for being repeated; 650 for not meeting the inclusion criteria. The remaining three articles were included and analyzed. The interventions consisted of practices with emphasis on rhythm and were conducted in groups. Risks of important biases were observed, such as lack of blinding in the allocation of participants and in the assessment of outcomes, as well as incomplete data for some outcomes.

**Conclusion:**

Overall, the results showed no evidence of efficacy of music-based interventions for cognitive outcomes in PD.

## Introduction

Parkinson’s disease (PD) is the second most common neurodegenerative disease in the world (Poewe et al., 2017). It is estimated that in 2030 there will be between 8.7 and 9.3 million cases of PD in the five most populous nations of Western Europe and in the 10 most populous nations in the world, entailing a significant and growing global financial and social burden (Asadpoordezaki et al., 2023). The main risk factor for PD continues to be age, so this trend is expected to continue as the global population ages (Caulfield et al., 2023). Its action results from the progressive loss of dopaminergic neurons in the substantia nigra pars compacta. Although the cause of dopaminergic loss is idiopathic, it is known that environmental and genetic factors—such as exposure to some toxic chemicals and mutations in specific genes—are part of its etiology (Simon et al., 2020). The growing number of studies on the subject encompasses a wide spectrum of research lines. Some are about biomarkers for cognitive impairment, clinical phenotypes, genetic predispositions, and pathophysiological pathways, for example. This demonstrates the heterogeneity of PD and represents challenges for future studies and implications for clinical practice (Wüllner et al., 2023).

It is motor symptoms that characterize the diagnosis of PD; however, it is known that cognitive deficits (and other non-motor symptoms) may be prodromal symptoms—such as executive function and working memory—that have been observed in people at risk of developing PD (Chahine et al., 2016). According to (Baiano et al., 2020), 40% of people with cognitive disorders in PD will develop mild cognitive impairment (MCI) over time. With the deterioration of cognitive functions and disease progression, 83% will present dementia after 20 years of diagnosis (Hely et al., 2008). The pathogenesis of cognitive disorders in PD is broad and still debated among researchers. Fang et al. (2020) address neurochemical alterations in the dopaminergic, cholinergic systems, as well as other neurotrophic factors as possible origins of cognitive deficits in PD.

Non-pharmacological interventions for the treatment of cognitive deficits in PD found in the literature are diverse. In recent studies, physiotherapy, occupational therapy, neuropsychology, dance, games, computer programs, music-based interventions, and brain and cranial stimulation interventions are found (see Sanchez-Luengos et al., 2021; Lawrence et al., 2017; Leung et al., 2015).

Music-based interventions are characterized by the use of music and/or musical elements as a therapeutic tool in various populations and for different outcomes (Loui, 2020). They are interventions that are easy to adhere to for patients of various ages and are considered low risk (Robb et al., 2011)—despite the risks of iatrogenic effects if not used properly (Murakami, 2021). The term “music-based interventions” is used in this study in a broad sense, including studies of music therapy.

Neurobiological bases may offer a pathway for understanding the effects of musical practices on cognition in individuals with PD. Seidler et al. (2010), coined the term Supply and Demand Framework (SDF) to explain a mechanism applied to age-related changes in motor control. According to the authors, there is a higher demand for cognitive processes to assist in motor control in older adults due to structural declines in motor cortical regions, cerebellum, and basal ganglia, along with neurotransmitter reductions. At the same time, the prefrontal cortex and anterior corpus callosum undergo degradation, which reduces attentional capacity and other relevant cognitive resources. The authors also state that areas associated with high levels of cognitive processing are acted upon by the dopaminergic system. The basal ganglia-thalamocortical (GBTC) and cerebellar-thalamocortical (CTC) networks, collectively, contribute to the generation and execution of movement. The disruption of the GBTC network in PD occurs due to dopamine reduction (Nombela et al., 2013). According to Zgaljardic et al. (2003), this dopaminergic depletion promotes important deficits in cognitive abilities in people with PD, from the disruption of front striatal circuits. In addition, areas such as the ventral prefrontal cortex, parietal, temporal, occipital, and basal ganglia are involved, reported in neuroimaging studies (Lopes et al., 2017; Mak et al., 2015).

Thus, Lesiuk et al. (2018), suggest improvements in cognitive performance or mitigation of potential cognitive deficits through musical training for fine motor skills, based on the strengthening of the cortico-cerebellar pathway promoted by these practices. The aforementioned strengthening process refers to long-term potentiation (Bliss & Gardner‐Medwin, 1973), a mechanism of synaptic neuroplasticity that is directly involved in cognitive functions such as learning and memory. Thus, the neuroplasticity promoted by musical practice may result in structural and functional changes in synaptic connections in sensorimotor and cognitive networks (Chatterjee et al., 2021).

Another factor that may be considered in this model is neurochemistry. Deficiency in dopamine production is characteristic of PD. Additionally, the significant correlation between music and the dopaminergic system is already known (see Chanda & Levitin, 2013). Thus, music may activate the limbic system by promoting feelings of pleasure, as well as facilitate dopamine release and improve adherence to long-term treatment interventions (Lesiuk et al., 2018), which may translate into more effective interventions.

Considering that interventions on the subject often consider the rhythm element of great relevance, it is important to consider rhythmic entrainment processes (the process that governs the dynamic alignments of the auditory and motor domains) and synchronization (stable maintenance of time during auditory-motor alignment), which are frequently addressed in the context of sensorimotor rehabilitation (Moumdjian et al., 2018; Thaut et al., 2015). These processes form the explanation of how auditory rhythmic stimuli promote motor organization and control. For example, the effects of this stimulation may be attributed to the model of temporal prediction and timing associated with the auditory stimulus. Thus, the authors suggest that auditory rhythmic stimulation helps patients recruit the CTC network, as a compensatory mechanism, which extends to supplementary motor areas (SMA) and frontal cortices (Nombela et al., 2013), promoting entrainment for motor processes under which there is a significant cognitive demand for execution (Seidler et al., 2010).

The pioneering works of Pacchetti et al. (1998) and Thaut et al. (1996) contributed to the development of the field. Pacchetti et al. (1998), evaluated emotional, motor, and quality of life outcomes in a prospective study describing methods of active music therapy. Thaut et al. (1996), presented the use of the Rhythmic Auditory Stimulation (RAS) technique for gait rehabilitation in people with PD. However, music-based interventions for cognition in PD are scarce (see Sotomayor et al., 2021; Raglio, 2015). Consequently, this implies few systematic reviews on the topic. For example, in a search for systematic reviews of studies on music, cognition, and PD, only the study by Sotomayor et al. (2021) was found, which does not limit its search criteria to randomized controlled trials or solely cognitive outcomes. Therefore, by considering randomized controlled trials in its criteria, this review aims to fill a gap in the literature, in addition to offering theoretical and practical subsidies for clinical practice in neurological rehabilitation. The objective of this study is to analyze the effectiveness of music-based interventions for cognitive outcomes in PD.

## Methods

This review was planned and conducted following PRISMA guidelines (Preferred Reporting Items for Systematic Reviews and Meta-Analyses), Cochrane Collaboration (Cumpston et al., 2019), and registered on the PROSPERO platform under the ID CRD42022332613.

### Eligibility criteria

The characterization for eligibility was based on the items used to define the research question. These items are: population, intervention, comparator, outcome, and study design (PICOS) (Amir-Behghadami & Janati, 2020).

Included studies met the following inclusion criteria: (1) individuals with idiopathic Parkinson's disease, (2) music-based interventions or music therapy, (3) assessment of cognitive outcomes, (4) a control group for comparison, and (5) a randomized controlled design. Studies without the descriptors in the title or abstract, studies with incomplete results, and studies in progress were excluded. There was no limitation on the publication date or language of the study. All searches were conducted in the English language.

The search for studies occurred in five databases with specific filters applied: PubMed/MEDLINE (“Title/Abstract”, “Randomized controlled trials”), PsycInfo (no filter), Scopus (“Title/Abstract/Keyword”, “Article”), Web of Science (“Articles”), and Cochrane (“Title/Abstract/Keyword”, “Trials”) on May 16, 2022. For the definition of the descriptors, a search was performed in the MeSH database and the following search strategy was used: “Parkinson’s Disease” OR “Parkinson Disease” OR “Idiopathic Parkinson’s Disease” AND “Music-Based Intervention” OR “Music Therapy” OR “Music Training” OR “Auditory Stimulation” OR “Music” OR “Rhythm” OR “Rhythmic*” AND “Cognition” OR “Cognitive”.

### Study selection

Two review authors independently reviewed the titles and abstracts of the articles retrieved from the databases. The articles were allocated in the Rayyan software (Amir-Behghadami & Janati, 2020) where duplicates were excluded. Subsequently, articles that did not meet the eligibility criteria as suggested in the PICOS strategy were excluded. Potentially relevant articles were retrieved for full-text reading and analysis. There was no disagreement between the authors in the classifications that required a third-party judgment.

### Data extraction

The following data were extracted: authors’ names, publication years, objectives, sample size and characteristics, type of intervention, tests used, and results. The two review authors independently reviewed the data according to the search strategy. There was no disagreement between the authors in the classifications that required a third-party evaluation. There was no missing data that justified contacting the authors.

### Risk of bias

Risk of bias assessment followed guidelines from the Cochrane Handbook for Systematic Reviews of Interventions version 6.3 (Higgins et al., 2011) and was performed in RevMan software version 5.4.1. Each article was assessed for selection biases (randomization and allocation), performance biases (blinding of participants and professionals), detection biases (blinding of outcome assessors), attrition biases (incomplete outcome data), reporting biases (selective outcome reporting), and other sources of biases.

## Results

### Search results

**Fig. 1:**
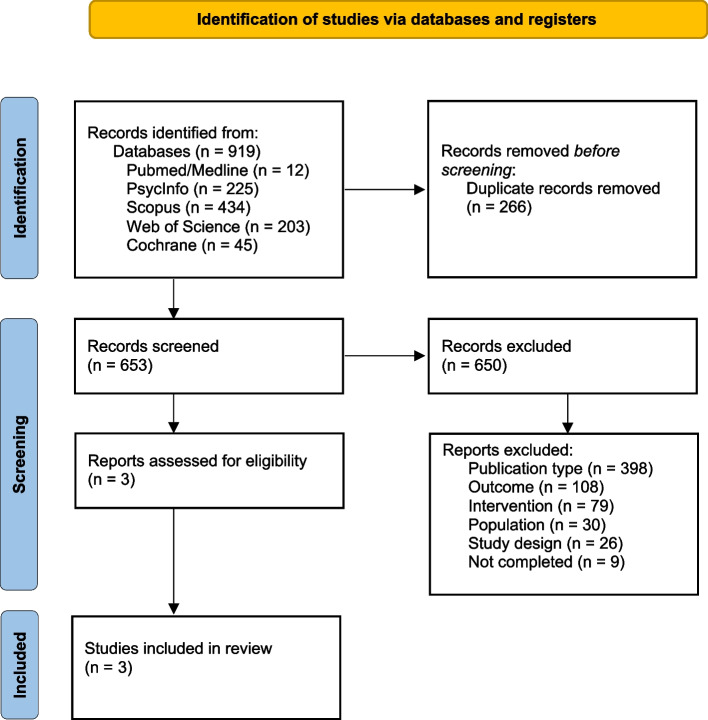
Search flowchart

In Fig. 1 (flowchart), it is possible to see all the data related to the search results. A total of 919 articles were identified. Of these, 266 were removed because they were duplicates. Of the 653 that went through the eligibility criteria stage, 650 were excluded, with the main reason being the wrong type of publication (*n* = 398), leaving only 3. These 3 were read in full and kept for meeting the stipulated criteria.

### Study characteristics

Table 1 shows information about participants, interventions, evaluated outcomes, and other study characteristics. In total, the three studies had 76 participants. The largest sample was Pohl et al. (2020), with 46 participants, and the smallest, with 12 participants, was Kim and Park, (2021). The publication period of the studies was between 2013 and 2021. The overall average age was 67 years. The maximum intervention period was 12 weeks, with three sessions per week (Kim & Park, 2021), and the minimum, was 6 weeks with two sessions per week (Pohl et al., 2013).

**Table 1:** Characteristics of the included studies

**Author**	**Year**	**Objective**	**Sample**	**Intervention**	**Results**
Jin-Kyoung Park, Soo Ji Kim	2021	To evaluate the effects of a drumming intervention with rhythmic cues on upper extremity motor control and attentional control in patients with PD.	Twelve participants, aged 40–60 years, with a diagnosis of a PD in stages I to III on the Hoehn and Yahr scale. Six participants in the intervention group.	Three sessions per week, for 12 weeks, in a group, lasting 50 min each session.	There were no statistically significant differences for cognitive outcomes.
Petra Pohl, Ewa Wressle, Fredrik Lundin, Paul Enthoven and Nil Dizdar	2020	To evaluate a group musical intervention in patients with PD.	Forty-six participants aged eighteen years, with PD at stages I to III on the Hoehn and Yahr scale. Twenty-six in the intervention group .	Two sessions per week, for 12 weeks, in group, lasting 1 h each session.	There were no statistically significant differences for cognitive outcomes.
Petra Pohl, Nil Dizdar, and Eva Hallert	2013	To evaluate the feasibility of the Intervention “The Ronnie Gardiner Rhythm and Musi Method”.	Eighteen participants diagnosed with PD. Twelve in the intervention group.	Two sessions per week, for 6 weeks, in a group, lasting 1 h each session.	There were statistically significant differences for learning and episodic memory, language and inhibitory control.

### Risk of bias

**Fig. 2:**
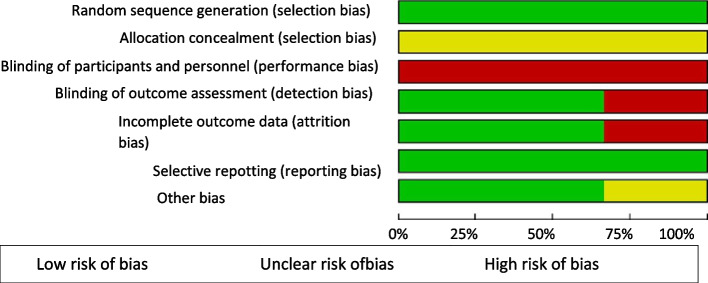
Risk of bias graph

**Fig. 3:**
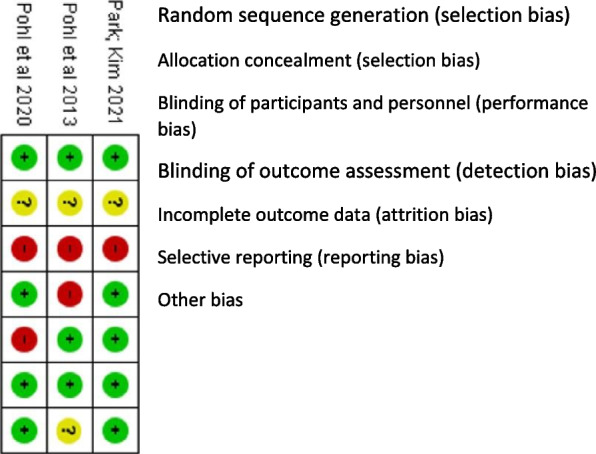
Risk of bias summary

In Figs. 2 and 3, the results for risk of bias assessment are presented. All studies met the first criterion for selection bias (random sequence generation), reporting bias (no selective outcome reporting), and the criterion for other sources of bias. None of the studies were clear about allocation concealment (insufficient information), generating an unclear risk of bias. Regarding performance bias, all studies presented a high risk of bias given the absence of blinding of professionals regarding the intervention. In one study (Pohl et al., 2013), no blinding of the outcome assessor was reported, generating a high risk of detection bias. In the study by Pohl et al. (2020), a high risk of attrition bias was identified due to missing data from some outcome measures, a problem reported by the authors themselves in their article. Regarding other sources of bias, Kim and Park (2021) declared no conflict of interest and reported no funding for the research. Pohl et al. (2020) reported a funding source for the study by the Ostergotland County Council and Department of Neurology, Linköping University Hospital, Linköping, Sweden, but declared no conflict of interest. Pohl et al. (2013) declare a possible conflict of interest due to the participation of a Ronnie Gardiner method practitioner in the study. However, they report that she remained blinded during the outcome assessments and participant interviews. The other authors of the study declare no conflict of interest and funding from Region Östergötland, Henry and Ella Margareta Stahls Foundation, Tornspiran Foundation, Neuro Sweden, Swedish Parkinson’s foundation, and Linköping University Hospital Research Fund.

### Outcome analysis

A total of 76 participants were evaluated for cognitive functions before and after interventions. Table 2 provides the specific information for each study. In Kim and Park (2021), the following outcomes were evaluated: attention and processing speed (K-TMT-e_A), cognitive flexibility (K-TMT-e_B), and two measures of inhibitory control (KST_WR_Time; KST_CR_Time). None of the outcomes showed statistically significant results (*p* = 0.786; *p* = 0.903; *p* = 0.808; *p* = 0.100; respectively).

**Table 2:** Tests, outcomes and results of included studies

	**Tests used**	**Evaluated outcomes**	*P*-value*
Kim and Park (2021)	Korean Trail Making Test A (K-TMT-e_A)	Attention and processing speed	0.786
	Korean Trail Making Test B (K-TMT-e_B)	Cognitive flexibility	0.903
	Korean Stroop Test Word reading Time (KST_WR_Time)	Inhibitory control	0.808
	Korean Stroop Test Color Reading Time (KST_CR_Time)	Inhibitory control	0.100
Pohl et al. (2020)	Montreal Cognitive Assessment Scale (MoCA)	Global cognitive function	0.347
	Text Immediate Recall	Immediate recall	0.126
	Text Delayed Recall	Delayed recall	0.643
	Stroop Color Word Test (s)	Inhibitory control	0.848
	Symbol Digit Modalities Test (no)	Attention and processing speed	0.064
Pohl et al. (2013)	Text Recall Test	Learning and episodic memory	**0.036**
	Symbol Digit Modalities Test (no)	Attention and processing speed	0.753
	Clox and Cube	Visuospatial functions	0.287
	Naming 30 items	Language	**0.033**
	Stroop Color Word Test (s)	Inhibitory control	**0.007**
	Parallel Serial Mental Operations (PaSMO)	Executive functions	0.054

Bold = statistically significant results

^*^Values referring to time x group interaction

In Pohl et al. (2020), global cognitive function (MoCA), immediate and delayed recall, inhibitory control, attention, and processing speed were evaluated without statistically significant differences (*p* = 0.347; *p* = 0.126; *p* = 0.643; *p* = 0.848; and *p* = 0.064, respectively).

In the study by Pohl et al. (2013), statistically significant results were found for learning and episodic memory, language, and inhibitory control (*p* = 0.036, *p* = 0.033, and *p* = 0.007, respectively). However, for attention and processing speed, visuospatial functions, and executive functions, no statistically significant results were found (*p* = 0.753, *p* = 0.287, and *p* = 0.054, respectively).

## Discussion

The aim of this review was to analyze the effectiveness of music-based interventions for cognitive outcomes in Parkinson’s disease. Overall, the results showed no evidence of efficacy of music-based interventions for cognitive outcomes in PD.

An important characteristic of the evaluated studies is that cognitive outcomes were considered secondary. This condition is relevant because secondary outcomes are treated with less importance in research (Zarin et al., 2016). That is, the fact that interventions were planned with other outcomes as primary may have influenced the results for cognitive outcomes. In the case of Parkinson’s disease, non-motor outcomes are expected to be evaluated as secondary, after all, PD is characterized for diagnostic purposes by motor symptoms (Balestrino & Schapira, 2020). In Pohl et al. (2020), for example, other outcomes such as balance and quality of life were reported with good results; freezing of gait and mobility did not improve. In Pohl et al. (2013), motor function and quality of life showed improvements in participants. In the study by Kim and Park (2021), measures of motor control and motor response time showed important improvements after intervention. It is important to highlight that the study by Pohl et al. (2013) is characterized as a feasibility study of an intervention method (The Ronnie Gardiner Rhythm and Music Method). Thus, the study concludes that the participants’ adherence was high and that the chosen evaluation tools are adequate for future studies with larger samples.

These results are supported in the literature. Spina et al. (2016), conducted a randomized controlled trial aimed at evaluating motor and non-motor outcomes in PD through music therapy. After 24 weeks of intervention, with one 90-min session per week and pre- and post-intervention assessments, participants were evaluated with a follow-up of 6 more months after the end of the intervention. The outcomes of verbal fluency, attention and processing speed, immediate and delayed recall, a measure for inhibitory control, and quality of life are reported with improvements in the first post-intervention assessment. However, the effects decreased in the last assessment, suggesting that the benefits decrease over time and that continuation of the intervention would be indicated. Other cognitive outcomes such as executive functions, cognitive functioning, did not show gains. An important limitation of the study is the sample size used. The risk of bias may be high due to the absence of relevant information such as the method of randomization, allocation, and blinding of outcome assessment.

Bugos et al. (2019), conducted a group piano training for people with PD and compared the cognitive outcomes with a control group. The intervention consisted of three hours of piano training per day for 10 days. The result indicated an improvement in inhibitory control in the intervention group compared to the control group. However, no benefits were found in other outcomes such as auditory information processing speed, information processing speed, motor speed in the cognitive flexibility test, verbal fluency, and attention. The main limitations of the study were the intervention time, sample size, and absence of a randomized controlled design.

Roesch et al. (2021), conducted a study on rhythmic interventions for cognition in PD. The objective was to compare two different rhythmic interventions: rhythmic speech-language rherapy (rSLT) and rhythmic balance-mobility training (rBMT). Interventions were performed three times a week for 4 weeks, which was characterized by the authors as intensive therapy. The group that received rSLT showed improvements in working memory and language. However, other cognitive outcomes such as attention, visuoconstructive function, and executive functioning did not improve. The main limitations of the study were the sample size, the duration of the intervention period, and the risks of biases due to non-randomization of participants.

Several limitations may be pointed out in the included studies. The first one refers to the considerably small sample sizes. In Pohl et al. (2013), this limitation is pointed out by the authors who suggest future research with a sample of 90 participants, assuming a drop-out rate of 20%. Another point is the lack of clear data on the allocation method of participants, which generates bias and decreases the methodological quality of the three studies. Incomplete outcome data (in Pohl et al., 2020) and lack of blinding in outcome assessment (in Pohl et al., 2013) also weaken the methodological quality. The lack of blinding of participants and applicators in studies of this nature is a normal characteristic. Therefore, despite the high risk of bias classification, this risk is considered an intrinsic limitation to the type of intervention. The group intervention may also be considered a limiting factor of the results, besides the absence of clinical implications and follow-up evaluation (only in the study of Pohl et al., 2020, a follow-up evaluation was performed).

This review presents some limitations. The search results showed a very low number of randomized controlled studies covering the researched topic. This result may be compared to the study by Sotomayor et al. (2021), who conducted a systematic review on music therapy for PD, considering the period from 2015 to 2020, and found only 4 intervention studies for cognitive outcomes, with only one (Spina et al., 2016) being randomized controlled. This is certainly a concerning point, since studies with this design are essential for building a consistent body of evidence for intervention in PD (Spieth et al., 2016). Another limitation observed is that the presented results of the studies make clear the lack of homogeneity in the type of intervention, and with results for cognitive outcomes being very varied.

The growth of research on the effect of music on development or rehabilitation in various pathologies is remarkable (Li et al., 2021). However, the various elements that constitute music and are used as intervention tools, end up leading to various different terms (e.g., “music therapy”, “music-based interventions”, “auditory rhythmic stimulation”, “musical training”, “piano training”, “auditory rhythmic cues”, “rhythmic interventions”, “music-supported therapy”), which may make it difficult to search for studies in systematic reviews. This condition, however, is intrinsic to the nature of musical practice, which (Loui, 2020) classifies as a complex intervention, that is, with multiple interacting components.

As recommendations for future studies, following guidelines from health research organizations is recommended. For example, Cuschieri (2019) cites the UK Medical Research Council (MRC), which recommends a model for the development and evaluation process of complex interventions; and also CONSORT (Consolidated Standards of Reporting Trails), considered an essential tool for conducting intervention studies such as randomized controlled trials. In the USA, the Food and Drug Administration (FDA) establishes a four-phase guide for conducting clinical trials (Loui, 2020). Study reporting may also follow the recommendation of Robb et al. (2011)—Reporting Guidelines for Music-based Interventions—who developed a guide to improve transparency and specificity of music-based interventions.

This systematic review has important implications for clinical practice. Knowledge about music and cognition in PD also opens up possibilities related to motor symptoms that are cardinal in PD, such as gait (Sousa et al., 2021). In this sense, by understanding the association between cognitive and motor skills for good gait performance, musical interventions can configure a practice in which interdependent skills are integrated. For example, inhibitory control as a skill that mediates gait performance and the appropriate temporal processing offered by rhythm for gait rehabilitation (Naro et al., 2023; Buard et al., 2019). Thus, future research can explore cross-sectional designs to identify how motor, perceptual and cognitive skills are related in the context of musical practice, in addition to randomized controlled studies that evaluate the effectiveness of interventions. Regarding dementia, a condition related to the evolution of cognitive impairment in PD, studies show that interactive musical activities can result in a reduction of behavioral and psychological signs that aggravate the condition. This occurs from cognitive stimulation (Shirsat et al., 2023) and well-being related to musical pleasure (Belden et al., 2023).

## Conclusion

The objective of this review was to analyze the effectiveness of music-based interventions for cognitive outcomes in Parkinson’s disease. Overall, the results showed no evidence of efficacy of music-based interventions for cognitive outcomes in PD. However, it should be considered that in PD and other degenerative conditions, the effects of interventions may be underestimated given the progressive nature of the disease (Moumdjian et al., 2017). For future intervention studies, it is recommended to use larger samples, follow-up evaluations, and a randomized controlled trial design that minimizes biases. The proposed recommendations aim to encourage future research with greater methodological rigor and more clinical implications for the PD population.

## Data Availability

The authors confirms that all data generated or analyzed during this study are included in this published article.
